# Clinical Lectures in the Ophthalmic Department of the Cook County Hospital

**Published:** 1874-11-01

**Authors:** F. C. Hotz


### CLINICAL LECTURES IN THE OPHTHALMIC DEPARTMENT OF THE COOK CO. HOSPITAL.

By F. C. Hotz, M.D.

*Reported by F. C. Winslow, M. D., House Surgeon.*

GENTLEMEN: In presenting a course of clinical instruction this winter it shall be my aim to select such cases for your observation as you will be most likely to encounter in the course of ordinary practice; and believing it to be the duty of every physician to understand thoroughly all the diseases and accidents incident to the external eye, which constitute by far the majority of cases which will apply to you for treatment, I shall confine myself mainly to cases of this character.

The subject I have chosen to present to you to-day is Blennorrhœal Conjunctivitis.

This disease, while it is liable to attack persons of any age, is certainly much more frequent in new-born children, constituting the *blennorrhœa neonatorum* of the authors. Hence the necessity of each practitioner being fully acquainted with the affection, not only because it is to him alone that the parents look for advice at this critical period, but because it is emphatically true that delays are dangerous and ignorance and hesitation are too often the means of consigning the helpless infant to a life of miserable darkness.

The disease may be caused by direct contagion, by exposure to a glare of light, to the continued irritation caused by foreign bodies, and in fact by a variety of causes.

The onset of the disease is marked by pain in the eye, a sensation of heat, and if the conjunctiva be examined it will be found to be unusually dry, and instead of the pale or pinkish hue of health, the vessels are fully injected, giving the membrane a scarlet appearance. This stage of the disease is speedily followed by a change in the external appearance of the lid, which becomes enormously swollen, while the skin covering its outer surface is red and glossy, owing to the infiltration into the cellular tissue beneath. The lid is soft, however, and easily everted, and I may mention in passing, that this constitutes one of the differential points between the affection under consideration and a diphtheritic inflammation. In the latter the lid feels hard, almost cartilaginous, and eversion is well-nigh impossible without inducing anæsthesia.

Upon raising the lower margin of the upper lid, which usually overlies the lower lid, a copious discharge of clean healthy pus is poured forth. This we gently remove with a soft sponge and lukewarm water, and expose the membrane for inspection.

Concerning this operation for the removal of the pus, never permit the use of anything but pure water slightly warm. Many mothers are in the habit of using for this object the secretion from the mammary gland. This should be strenuously discountenanced, as being in no way suitable for the purpose. The portion of the conjunctiva lining the lid is thrown into large folds, tender, red, and disposed to bleed at the slightest touch, while the ocular portion is infiltrated with serum to such an extent as to bulge forward immediately on raising the lid, thus causing the margin of the cornea to appear depressed.

The result of the disease is of course a matter of great importance. It may terminate in various ways; ulceration of the cornea is unfortunately a too common complication, such an accident resulting in a partial or total loss of vision. The disease in some of its milder forms may run its course and subside, without permanent injury to any of the important structures, leaving granulations which are always extremely slow of removal.

I wish to say a word concerning the difference between the disease under consideration and other affections of the lids. The only disorder with which it is at all likely to be confounded is a diphtheritic inflammation of the lids, and the chief points of distinction between the two are these:

1. In the latter the lids are hard, almost cartilaginous, to the touch.

2. It is impossible to evert the lid without the use of an anæsthetic.

3. The mucous lining is not red, succulent, and liable to bleed at a slight touch, but is pale, anæmic, and covered with the characteristic exudation.

4. The discharge, instead of being of a thick, creamy, purulent character, is thin, turbid and watery.

The prognosis is regulated by the state of the cornea at the time of the first examination. If the cornea is clear the patient may be encouraged to hope for a favorable termination. But if there is a slight abrasion of the epithelium, the constant contact of the irritating secretion will surely develop ulceration, which will most likely be followed by opacity. Or the ulceration may be deep enough to cause perforation, which may be followed by hernia of the iris and anterior synechia.

Treatment: A prominent feature in the care of these cases is cleanliness. Each patient must have his own basin, towel and sponge, and the lids must be carefully cleaned, not three or four times daily, but as often as the secretion accumulates. For this purpose a sponge is the most suitable. A syringe is almost useless. It is entirely inadequate to the removal of the pus adherent to the roughened mucous membrane, and by everting the lid and using a sponge you obtain the additional advantage of being able to *see* what you are doing.

Abstain from the use of milk or teas in cleansing the lid. Nothing is so appropriate as pure warm water. In the œdematous condition which is sometimes present in the ocular conjunctiva, depletion is attained by scarification of the conjunctiva, i. e., superficial incisions radiating from the cornea. By this means you avoid retraction of the membrane and large cicatrices. As a local application experience shows that a solution of ag. nit. gr. xx, xxx or xl, to aq. dist. ℥j., according to the severity of the disease, is the best application; this must on no account be used through a syringe, but, carefully everting the lids, to prevent the solution coming in contact with the cornea, touch them with a camel's hair pencil dipped in the solution and then wash them with the brush until the water runs out clear.

CASE I.—A Swede, aged forty; third week of treatment; has had daily application of ag. nit. gr. xx, to ℥ j., and occasionally stronger. The œdema has disappeared from the lid and there is only a slight discharge of pus. He will receive an application only on the lower lids.

CASE II.—An Irishman, aged forty-eight; was taken a few days later than the other. The œdema of the lid has passed away, but the membrane covering the sclerotic still needs occasional scarification.

CASE III.—The most recent case. The lids are still puffy and succulent, and the discharges profuse. He will receive an application of ag. nit. gr. xxx, to ℥ j. once in twenty-four hours, or oftener if necessary.

